# Cognitive Training with Neurofeedback Using fNIRS Improves Cognitive Function in Older Adults

**DOI:** 10.3390/ijerph19095531

**Published:** 2022-05-02

**Authors:** Bianca P. Acevedo, Novia Dattatri, Jennifer Le, Claire Lappinga, Nancy L. Collins

**Affiliations:** Department of Psychological and Brain Sciences, University of California Santa Barbara, Santa Barbara, CA 93106, USA; novia@ucsb.edu (N.D.); jle@ucsb.edu (J.L.); clairelappinga@ucsb.edu (C.L.); ncollins@ucsb.edu (N.L.C.)

**Keywords:** cognitive training, fNIRS, cognitive function, prefrontal cortex

## Abstract

This study examined the effects of a 4-week cognitive training program with neurofeedback (CT-NF) among 86 healthy adults (M = 66.34 years, range 54–84) randomized to either a treatment (app-based ABC games) or control (Tetris) group. Participants completed seven cognitive assessments, pre- and post-intervention, and measured their cortical brain activity using a XB-01 functional near-infrared spectroscopy (fNIRS) brain sensor, while engaging in CT-NF. The treatment (ABC) group showed significant (pre/post-intervention) improvements in memory (MEM), verbal memory (VBM), and composite cognitive function, while the control group did not. However, both groups showed significant improvements in processing speed (PS) and executive function (EF). In line with other studies, we found that strength of cortical brain activity (measured during CT-NF) was associated with both cognitive (pre and post) and game performance. In sum, our findings suggest that CT-NF and specifically ABC exercises, confer improved cognition in the domains of MEM, VBM, PS, and EF.

## 1. Introduction

Neuroplasticity, or the dynamic tendency of neural networks in the brain to adapt to external challenges, is an innate trait considered to be most present during childhood. Although the developing brain demonstrates a capacity for neuroplastic growth, research from the past decade has cultivated a new appreciation for the neuroplastic potential persistent in aging individuals as well. In aging adults, this type of process, termed activity-dependent plasticity, has been found to be induced by intrinsic activities and may be capable of stimulating the brain’s aptitude to learn and remember, even at an older age. This finding has led to the development of a variety of cognitive training programs. 

Cognitive training (CT) programs are repeated and structured mental exercises designed for initiating and enhancing cognitive function. Since the emergence of CT, numerous studies focusing on its potential benefits have emerged. For example, research has examined CT’s effectiveness for mitigating symptoms associated with neurodegenerative disorders [1], for promoting neuroplastic processes [2], and for improving general cognitive function [3,4]. More specifically, one study of 11,470 participants (M age = 39 years old) assigned to engage in a 15 min CT program for 10-weeks showed significant improvements in memory, reasoning, and processing efficiency when compared to an active control (ACT) group tasked with completing crossword puzzles [5]. The effectiveness of CT has also been demonstrated when administered in non-traditional formats. For instance, a study involving adults ages 60 to 85 showed that when assigned to play one-hour of the video game “NeuroRacer” three times per week for four weeks, participants showed increases in sustained attention, working memory, and prefrontal cortical activity [6]. Although numerous studies have provided support for the cognitive benefits associated with engaging in CT, these programs have limitations such as requiring long-term commitment. Moreover, they often have limited adherence due to the lack of positive reinforcement and practical difficulties faced by individuals with cognitive impairments and dementia.

Neurofeedback (NF) is a technique where an individual regulates their own brain activity by measuring it and receiving a feedback signal in real time [7]. NF provides participants with reassurance and reinforcement, and thus it may also enhance performance and adherence. As such, NF (an operant conditioning approach) has been shown to be positively associated with targeted brain region activation [8]. Numerous studies have demonstrated that after undergoing NF training, participants were able to successfully up- or down-regulate their own brain activity [9,10]. Moreover, a review of 47 studies examining the effects of NF in healthy subjects showed that NF impacted working memory, mood, sleep quality, executive function, and attention [11].

Nevertheless, NF findings have been mixed—likely due to the variability in experimental approaches, learning strategies, training conditions, and individual differences in motivation and practice [11,12,13]. One reason for mixed findings may be due to the addition of NF to other protocols, as there is some evidence that NF may provide optimal benefits in combination with other CT approaches. For example, one study showed no improvements in executive function among participants engaging in NF training alone [9]. Thus, researchers have tended to use NF in conjunction with CT programs [10,14,15]. The findings suggest that combining CT with NF (CT-NF) results in superior cognitive improvements compared to applying either of the techniques alone. For example, in a study of 60 participants assigned to either CT alone, CT-NF, or an active control (ACT) condition; the CT-NF treatment group showed significant improvements in episodic memory, working memory, and attention compared to the CT-alone and ACT groups [16]. The CT-NF group also showed stronger brain activity, as measured with fNIRS (functional near-infrared spectroscopy), compared to the other two groups. Other studies have replicated these findings, confirming that the combination of CT with NF may be associated with greater cognitive benefits [17]. 

Thus, in the present study, we examined the effects of engaging in a four-week CT-NF program involving app-based games (treatment group) versus playing Tetris (active control group, ACT). For the NF component, participants were provided a XB-01 brain sensor device, which uses fNIRS technology to track blood flow changes in the frontal brain. Compared to other neuroimaging techniques, fNIRS is an ideal compromise as it provides a portable and lightweight brain monitoring method that is less sensitive to motion artifacts, does not impose significant physical restraints, and facilitates the measurement of brain activity while performing everyday tasks in real-time [18].

We selected Tetris as an ACT group because it involves complex mental activities, such as the use of visuospatial abilities and working memory. However, findings have been mixed regarding Tetris’ association with improved cognitive function. For example, a study examining 59 undergraduate students who played Tetris for four one-hour sessions, showed no significant improvements in cognition, specifically in spatial and perceptual skills (Study 1), or when compared to a group of 25 undergraduate students that did not play Tetris (Study 2) [19]. Nouchi et al. (2012) [20] further confirmed the viability of Tetris as an ACT group in their study involving 28 healthy older individuals (M age = 69 years old). Participants were instructed to either play Tetris or the video game “Brain Age” for 15 min per day, for at least five days per week, for four weeks. Despite all other factors being controlled for, only participants in the treatment group displayed enhanced processing speed and executive function. 

In contrast, some studies suggest that playing Tetris has some cognitive benefits. For example, one study testing the effects of video-game playing on novice older adults’ cognition (ages 75–91, M age = 75 years old) found that participants assigned to either play a video game or Tetris (for six, 90 min sessions administered over 2–3 weeks) showed significantly higher pre-post benefits in selective visual attention, compared to the ACT [21]. In another study of older adults (M age = 75 years old), those that played either a video-game or Tetris showed higher pre-post “Flow” ratings after six 90 min sessions, compared to a control group [22]. “Flow” is a deep sense of contentment, well-being, mastery, and heightened self-esteem [23]. In yet another study, significant pre-post increases in working memory were found among 18 adolescents with attention-deficit/hyperactivity disorder (ADHD) who were instructed to play Tetris (treatment) versus an alternate computerized cognitive exercise regime [24]. However, these same participants failed to show significant increases in sustained attention or any decrease in symptoms of their ADHD, post-trial. 

Thus, we examined the effects of CT-NF among a group of participants randomized to either engage in the “Active Brain Club” (ABC) application games involving a combination of cognitive training games (treatment), or the Tetris group (ACT). We hypothesized that CT-NF with ABC exercises would result in greater improvements in cognitive function, compared to one single program (Tetris). Moreover, we expected that the strength of right (r.) prefrontal cortex (PFC) activity during the CT-NF would be a reliable indicator of and positively associated with participants’ cognitive function and game performance. 

## 2. Methods and Measures

### 2.1. Procedure

Participants provided informed consent in accordance with the IRB procedures of the University of California, Santa Barbara (UCSB). Subjects were recruited via advertisements, flyers, listserv groups, independent living communities, and word of mouth in the Santa Barbara community. Recruitment flyers and emails provided a link to the study website (https://ectstudy.psych.ucsb.edu/ (accessed on 8 April 2021). The study website provided viewers with information about the study including eligibility criteria, procedures, a link to the study’s online screening form, and compensation. The study team reviewed the data from the online screening form, and once eligibility was confirmed, participants were sent a welcome email and links to complete the online consent form and the baseline survey. 

Next, participants completed a brief enrollment phone call with a member of the study team to review procedures. After the enrollment call, each participant was sent an e-mail with instructions explaining how to complete the online cognitive assessments and a package containing the study materials. Study materials included one XB-01 brain sensor device with a headband (NeU corporation, Tokyo, Japan), a tablet containing the ABC application (to be used for CT-NF), written instructions, and a log sheet. Next, participants completed a live, virtual, 45 to 60 min interactive training session, via Zoom, with 3 to 7 other study participants. In the training session, participants received a demonstration of how to use the XB-01 brain sensor, Android tablets, and either the ABC app or Tetris, depending on their group assignment. They also received detailed instructions and safety precautions. Participants were asked to engage in CT-NF for about 10–15 min per day, 5 to 7 days per week, for 4-weeks. 

### 2.2. Cognitive Assessments

Online cognitive tests have been shown to be reliable and accurate indicators of cognitive health and impairment and have yielded similar results in satisfaction and usability when compared to traditional paper-and-pencil tests [25,26,27,28]. Thus, we administered cognitive assessments with the online platform “CNS Vital Signs” (https://www.cnsvs.com/ (accessed on 14 April 2021). Five cognitive tests—the Verbal Memory Test, the Visual Memory Test, the Symbol Digit Coding Test, the Shifting Attention Test, and the Four-Part Continuous Performance Test—were administered to assess seven cognitive domains: memory (MEM), verbal memory (VBM), visual memory (VSM), processing speed (PS), executive function (EF), working memory (WM), and sustained attention (SA). The online cognitive tests were administered before (pre) and after (post) the 4-week CT-NF program and took about 30 min to complete. Upon completion, experimenters reviewed the score reports. If any individual test was scored as “invalid,” a code was sent to the participant to repeat only the test that was scored as invalid. This process was repeated until the score report returned as valid. The number of times that a re-score was sent was included in our analyses to weigh those who scored successfully after fewer tries more heavily. 

### 2.3. Questionnaires

Participants completed a battery of questionnaires prior to beginning the CT-NF protocol, including questions about demographics. At the end of each CT-NF week, they also completed a short weekly check-in survey including questions about their user-experience, mood, and life satisfaction. The user-satisfaction scale included 10 questions on a 1 to 10-Likert Scale asking about enjoyment of the CT, ease of use of the device and brain sensor, fun related to the CT, wanting to learn more about the CT, and any negative feelings such as anxiety, tiredness, skin sensitivities, and awkwardness using the devices. 

### 2.4. Participants

**Figure 1:**
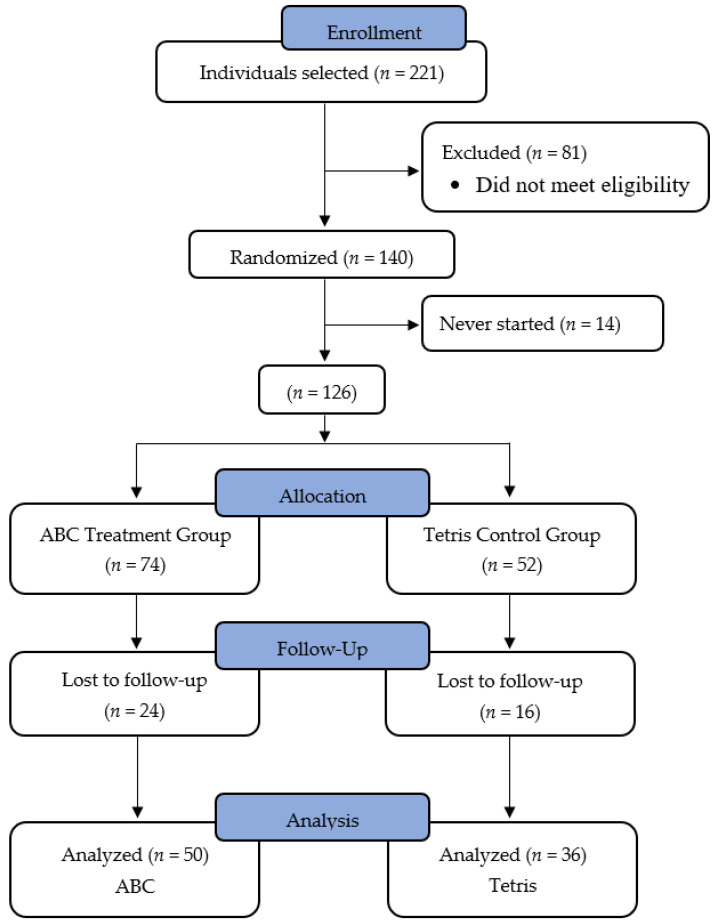
Flow chart of participant enrollment and attrition. 221 individuals initially enrolled in the study. After screening and withdrawals, a total of 86 participants (ABC: *n* = 50; Tetris: *n* = 36) remained in the final sample for analyses.

A flow chart showing enrollment and withdrawal statistics is shown in Figure 1. Of the participants that enrolled in the ECT study but withdrew: 54% stated personal reasons such as health issues, having to take care of a sick relative, not having enough time, or having unexpected travel; 23% stated they did not understand the devices or had technical difficulties; and the other 23% did not provide a reason for withdrawing or never began the study.

### 2.5. Treatment and Control Groups

#### 2.5.1. Cognitive Training Group

Participants in the CT-NF group engaged in 4-weeks of playing three different ABC games (Random Numbers, Endless Addition, and Forwards Order) three times daily. They were asked to wear the brain sensor while playing the games in order to measure their brain activity during training such that they received both a game score and a brain activity score at the end of each game. Daily training sessions lasted about 10 min per day. Participants were instructed to engage in the CT-NF for a minimum of five days per week, but they could also play every day if they desired.

#### 2.5.2. Active Control Group

Participants in the Tetris group were instructed to play three rounds of Tetris every day, resulting in approximately 10 min of daily training, similar to the CT group. They were also instructed to wear the brain sensor during training, and to record both their brain activity and game score after each game. Participants were instructed to engage in the CT-NF five to seven days per week. 

#### 2.5.3. Compliance

Compliance with the study protocol was assessed by the number of brain training days (BT): the number of days each participant completed the CT-NF program. Results showed that on average, participants completed 23 days (M = 22.64, SD = 4.11) of CT-NF. Moreover, a significant difference was found in BT days for the ABC (M = 23.58, SD = 4.17) versus the Tetris group (M = 21.07, SD = 3.56), t (75) = 2.71, *p* < 0.01. Thus, we controlled for mean number of BT days when conducting inter-group analyses. 

### 2.6. Data Acquisition and Analysis 

Our primary hypothesis was that the CT-NF treatment group (ABC) would show greater improvements in cognitive function compared to the active control (Tetris) group. As a first step in our data analytic procedures, we examined the effect of age on participants’ baseline cognitive scores. Results showed that age was negatively correlated with pre-intervention scores for MEM (r = −0.23. *p* < 0.05), VSM (r = −0.24, *p* < 0.05), PS (r = −0.29, *p* < 0.01), EF (r = −0.27, *p* < 0.05), and SA (r = 0.24, *p* < 0.05). Age was also negatively correlated with post-intervention scores for PS (r = −0.33, *p* < 0.01) and EF (r = −0.31, *p* < 0.01). Thus, we controlled for age in the analyses by computing partial correlations where appropriate and applying age as the control variable. We also examined gender differences in cognitive scores and found that males showed higher levels of pre-intervention VSM (M = 45.34) compared to females (M = 42.77, t (84) = 2.35, *p* < 0.05). Therefore, we examined cognitive test results with gender as a covariate, and the pattern of results remained the same. 

#### 2.6.1. Main Outcome Measures

Our main outcome measures were the seven cognitive tests, assessed both at pre- and post-intervention. First, we computed weighted scores for each test as outlined below (see Section 2.6.2). We then computed two “composite scores” summarizing the cognitive test assessments for all seven tests computed separately for pre- and post-intervention assessments. Finally, we calculated difference scores in weighted pre- and post-intervention cognitive scores for each of the seven cognitive measures (seven individual tests and one composite cognitive score).

#### 2.6.2. Cognitive Test Score Weighting

Each cognitive test was weighted according to the number of re-tests needed to complete the test successfully. If the participant completed the cognitive test successfully on the first try, the raw score was used. However, for each re-test given, the cognitive test was weighted by 0.8 to account for learning effects. All cognitive test analyses were carried out with the weighted scores. 

#### 2.6.3. Paired-Sample *t*-Tests

To examine intra-group differences in cognitive function, we conducted a series of paired-samples *t*-tests to assess significant differences in pre and post cognitive test scores by each individual test (MEM, VBM, VSM, PS, EF, WM, and SA) and for the composite cognitive score. 

#### 2.6.4. Multiple Regression Data Analysis

Multiple regression analyses were carried out to estimate correlations between major study variables. When appropriate, we also computed partial correlations to estimate associations between major study variables when controlling for age or gender. 

#### 2.6.5. ANCOVA Analyses

To examine intra-group differences in cognitive change scores, we conducted a univariate analysis of covariance (ANCOVA) for the change in scores (post-pre) for each of the seven cognitive tests and the composite score. The change score was entered as the dependent variable, “Condition” (ABC and Tetris) was entered as the independent variable; and age, number of CT-NF days, and pre-intervention cognitive score, were entered as the covariates.

## 3. Results

### 3.1. Descriptive Statistics

The sample was composed of 86 (65 females and 21 males) participants from ages 54 to 84 (M = 66.34 years). The majority of the sample was college-educated: 5% had an Associate’s degree, 38% had a Bachelor’s degree, 33% had a Master’s degree, 16% had either a professional degree or a doctorate, and only 17% reported having only a high school degree (2%) or some college education (15%). Approximately 54% of the sample was retired, 37% were employed (16% were employed full-time, 10% were employed part-time, and 11% were self-employed), 3% were homemakers, and 10% replied “other”. With respect to ethnicity, 88% of the sample was White, 6% were Hispanic/Latino, 5% were Asian, 2% were Black/African American, and 4% replied “Other.” Descriptive statistics for the study sample are reported in Table 1. There was a significant difference in age across the two groups; thus, we controlled for age in our analyses.

**Figure 2:**
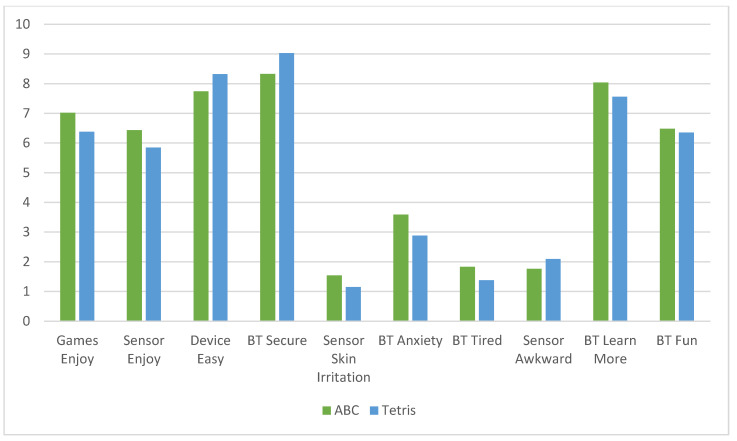
Cognitive training user-experience satisfaction ratings by group. Satisfaction ratings scored on a 10-point scale with 1 = low satisfaction and 10 = high satisfaction. Mean satisfaction was high for both the ABC (M = 7.93, SD = 1.17) and Tetris (M = 8.00, SD = 1.18) cognitive training groups.

Mean user-experience (UX) satisfaction ratings (10-items) showed similar scores for the ABC (M = 7.93, SD = 1.17) and Tetris (M = 8.00, SD = 1.18) groups, representing high-user satisfaction with the CT-NF program, regardless of treatment group. (See Figure 2).

### 3.2. Cognitive Outcomes 

Table 2 and Table 3 provide descriptive statistics and results from paired samples t-tests examining pre- to post-changes in cognitive function for the ABC (treatment) and Tetris (ACT) groups, respectively. The ABC group showed significant improvement in composite cognitive function, from pre- to post-intervention, as measured by the composite score of all cognitive tests (*p* < 0.01), while the Tetris group did not (*p* = 0.50). Paired-sample t-tests of the individual cognitive tests revealed that the ABC group showed significant improvements in MEM, VBM, PS, and EF (Table 2), while the Tetris group only showed significant improvements in PS and EF (Table 3).

**Figure 3:**
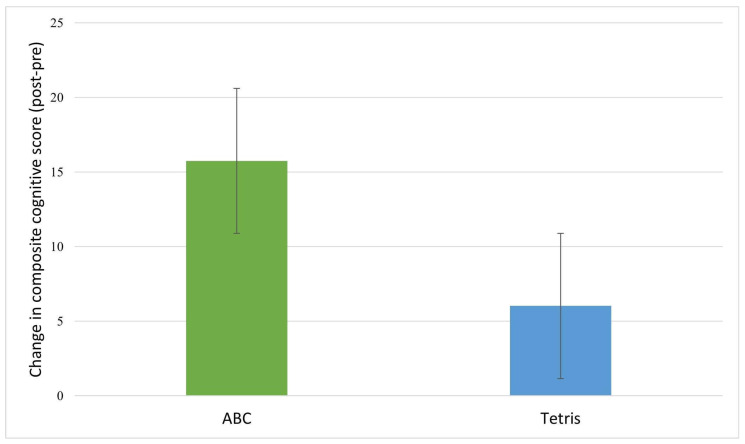
Composite cognitive function change scores by group. The ABC (treatment) group showed significant pre-/post-improvements in composite cognitive function; (*p* < 0.01). The control (Tetris) group did not show significant improvements in composite cognitive function (*p* = 0.50).

**Figure 4:**
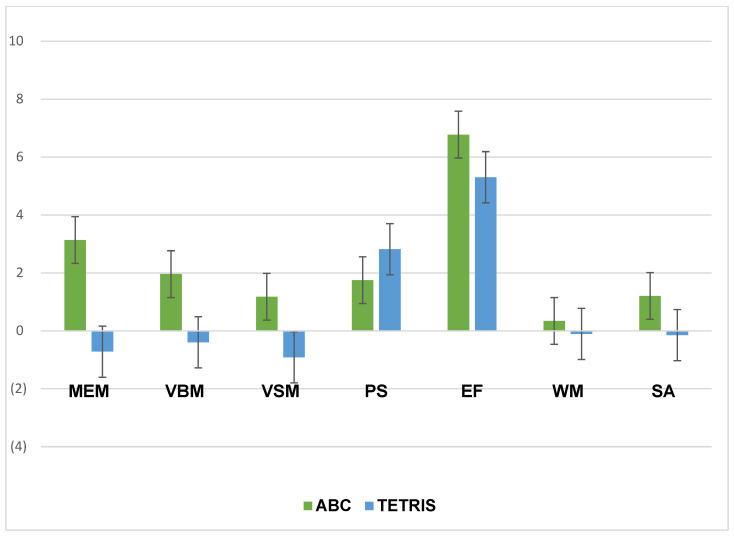
Individual cognitive test change score by group. The treatment (ABC) group showed greater improvements in memory (MEM), verbal memory (VBM), visual memory (VSM), processing speed (PS), executive function (EF), and sustained attention (SA) relative to the control (Tetris) group. WM = Working Memory.

Table 4 provides the mean change scores by group and results of ANCOVAs assessing between-group differences in cognitive score changes. Inspection of the cognitive change scores revealed that post-intervention, the treatment (ABC) group showed greater increases in composite cognitive function (Figure 3), memory, verbal memory, visual memory, executive function, and sustained attention relative to the control group (Figure 4). However, the between group differences were not significant. 

Mean change scores and ANCOVA results analyzing the relationship between treatment (versus control) group and change scores (post-pre) for composite cognitive function and each of the seven cognitive tests, while controlling for age, pre-intervention cognitive score, and number of cognitive training days.

Thus, we went on to investigate inter- and intra-group differences with a 2 × 2 ANOVA. Results showed significant within-subject effects of time, such that there was an improvement in composite cognitive function from pre (M = 306.36, SD = 45.14) to post-intervention (M = 317.42, SD = 43.33), F = 4.24, *p* < 0.05; as well as for PS from pre- (M = 45.70, SD = 9.35) to post-intervention (M = 48.02, SD = 9.44), F = 7.70, *p* < 0.01, and EF from pre- (M = 35.79, SD = 16.19) to post-intervention (M = 41.97, SD = 12.37), F = 33.35, *p* < 0.001. We also investigated inter-group differences in cognitive change scores for the composite cognitive score and individual cognitive tests with a series of ANCOVAs where the change score was the dependent variable; and age, pre-intervention cognitive score, and number of BT days were entered as covariates. No significant inter-group differences were shown (Table 4). 

### 3.3. Brain Activity Results 

**Figure 5:**
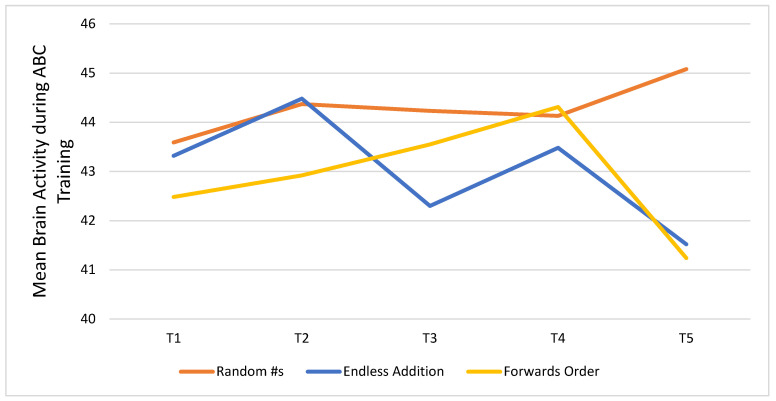
Trend in brain activity for every 5 days of cognitive training for the treatment (ABC) group, shown separately for each ABC game played.

Figure 5 shows trends in brain activity measured after every five days of participation in the CT-NF ABC program. Results are shown for each of the three games participants played: Random Numbers (RN), Endless Addition (EA), and Forwards Order (FO). Inspection of the plot showed that participants’ mean brain activity peaked at T5 for RN, and the change from T1 (43.59) to T5 (45.08) was significant (*t* = 1.91, *p* < 0.05). For EA and FO, brain activity peaked at T2 (44.48) and T4 (44.31), respectively, but the improvements were not significant relative to T1 EA: (M = 43.32) and FO (M = 42.48).

### 3.4. Correlations of Brain Activity with Pre-Intervention Cognitive Scores

**Figure 6:**
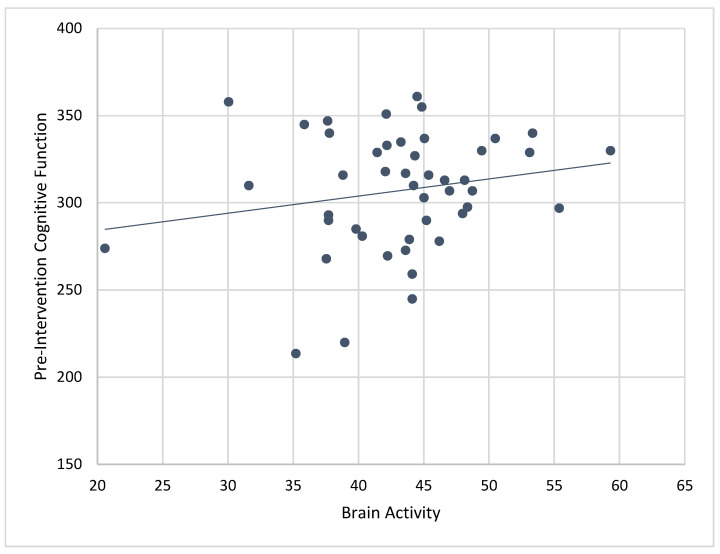
Correlation between pre-intervention composite cognitive scores and brain activity for the treatment (ABC) group. Composite cognitive function scores at baseline predicted mean levels of brain activity during cognitive training (r = 0.35, *p* < 0.01), and also when controlling for age (r_p_ = 0.41, *p* < 0.01).

Composite cognitive function scores at baseline were significantly associated with mean brain activity recorded while engaging in ABC games during the 4-week CT-NF program (r = 0.43, *p* < 0.01). See Figure 6. Correlations between brain activity and individual pre-cognitive tests showed significant associations for MEM (r = 0.35, *p* < 0.01), VBM (r = 0.35, *p* < 0.01), PS (r = 0.31, *p* < 0.05), EF (0.36, *p* < 0.01), WM (r = 0.28, *p* < 0.05), and SA (r = 0.35, *p* < 0.01). When controlling for age, correlations between baseline cognitive scores and mean brain activity were similar for composite cognitive function (r_p_ = 0.41, *p* < 0.01), MEM (r_p_ = 0.37, *p* < 0.01), VBM (r_p_ = 0.38, *p* < 0.01), EF (r_p_ 0.32, *p* < 0.05), WM (r_p_ = 0.28, *p* = 0.05), and SA (r_p_ = 0.32, *p* < 0.05). Only the correlation between pre-intervention PS and brain activity became non-significant when controlling for age (r_p_ = 0.25, *p* = 0.08).

We also examined correlations between baseline cognitive scores and brain activity measured while playing each of the ABC games during the 4-week CT-NF program. Significant correlations were only found for the RN game such that pre-intervention composite cognitive function (r = 0.43, *p* < 0.01), MEM (r = 0.35, *p* < 0.01), VBM (r = 0.33, *p* < 0.01), PS (r = 0.32, *p* < 0.01), EF (r = 0.36, *p* < 0.01), WM (r = 0.30, *p* < 0.05), and SA (r = 0.34, *p* < 0.01) predicted mean levels of brain activity while playing RN.

### 3.5. Correlations of Brain Activity with Post-Intervention Cognitive Scores

**Figure 7:**
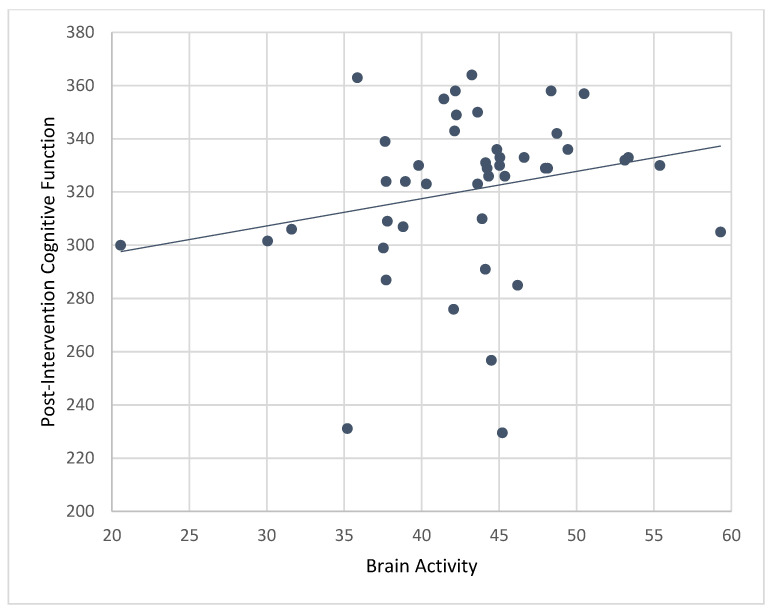
Correlation between post-intervention composite cognitive function and brain activity for the treatment (ABC) group. Mean levels of brain activity during ABC training were significantly associated with post-treatment composite cognitive function (r = 0.45, *p* < 0.001).

Results from bivariate correlations showed that mean levels of brain activity while playing ABC games during the CT-NF program were significantly associated with post-intervention composite cognitive function (r = 0.45, *p* < 0.001; Figure 7), PS (r = 0.37, *p* < 0.01), EF (r = 0.37, *p* < 0.01), and SA (r = 0.29, *p* < 0.05).

Controlling for age and pre-treatment cognitive scores, correlations between brain activity and composite cognitive function (r_p_ = 0.26, *p* < 0.05) remained significant, but not for PS (r_p_ = 0.18, *p* = 0.11), EF (r_p_ 0.16, *p* = 0.15), and SA (r_p_ 0.02, *p* = 0.89). Results for individual games only showed significant associations for RN such that mean levels of brain activity while playing RN (during the 4-week CT-NF program) predicted post-intervention composite cognitive function (r = 0.48, *p* < 0.001), PS (r = 0.37, *p* < 0.01), EF (r = 0.38, *p* < 0.01), and SA (r = 0.30, *p* < 0.05).

### 3.6. Correlations of Brain Activity with Game Scores

**Figure 8:**
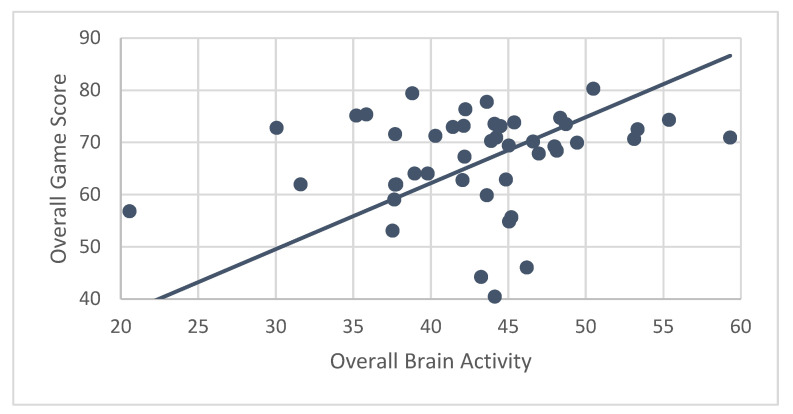
Correlation between performance (game scores) and brain activity for the treatment (ABC) group. Mean levels of brain activity during ABC training were significantly associated with performance on the ABC games (r = 0.84, *p* < 0.001).

A secondary hypothesis was that performance (measured by ABC game scores) would be correlated with brain activity. Results showed that ABC participants’ mean brain activity while engaging in CT-NF was significantly associated with their performance (r = 0.84, *p* < 0.001) (Figure 8). Even after controlling for age, correlations between brain activity and game score were nearly identical (r_p_ = 0.83, *p* < 0.001).

Correlations for individual games only showed a significant association for mean game score earned for playing RN with average brain activity while playing RN (r = 0.77, *p* < 0.001). However, after controlling for age, the correlation became non-significant (r_p_ = 0.02, *p* = 0.88). EA and FO game scores did not show significant associations with brain activity, even after controlling for age. 

## 4. Discussion

This study examined the effects associated with participation in a 4-week CT-NF program among older adults assigned to either a treatment (ABC) or ACT (Tetris) group. Results showed that engaging in the treatment (ABC) CT-NF program resulted in significant pre/post improvement in cognitive function, calculated as a composite score of seven cognitive tests for memory (MEM), verbal memory (VBM), visual memory (VSM), working memory (WM), processing speed (PS), executive function (EF), and sustained attention (SA). Individual cognitive test results also showed significant improvements in MEM, VBM, PS, and EF for the treatment group, while the control group only showed improvements in PS and EF from pre- to post-intervention. 

These results are especially promising for three reasons. First, it is widely known that cognitive function declines with age [6,29,30,31]. Therefore, it is of exceptional note that the current sample of older individuals demonstrated substantial improvements in several measures of cognitive function after participating in 4-weeks of CT-NF involving ABC app-based games. Secondly, our results align with those found in a previous study of 28 elderly individuals, which showed significant enhancements in EF and PS in response to CT [20]. Third, our results support current literature suggesting that brain activity measured during CT can reliably predict cognitive functioning and performance. 

In the present study, we found a significant association between r. PFC activity with pre- and post- intervention composite cognitive function (for the ABC treatment group). Interestingly, the results of the individual cognitive tests showed a significant association between post-intervention PS, EF, and SA with the activation of the r. PFC during CT-NF. These results are in line with the established role of the PFC in cognitive control and EF [32]. Our findings also support those published by Nouchi et al. (2020) who found that the activation of the bilateral PFC was associated with improvements in PS among a sample of 72 adults [33], as well as other studies showing similar effects. For example, one study assessing cognition and brain activity with fNIRS among 19 healthy adults (M age = 32 years old) found significant associations between memory recall and the activation of the bilateral PFC [34]. Another study with adults ages 20 to 30 showed that engagement in CT-NF resulted in the strongest levels of brain activity (measured with fNIRS), and subsequently, in improvements in cognitive function compared to the CT-alone and ACT groups [16]. Herein, we found significant associations between r. PFC activation with both pre- and post-intervention cognitive function among older individuals, suggesting that the levels of brain activity measured during CT may reliably predict cognitive function. 

The present study also adds to the body of work on the cognitive effects of engaging in CT-NF. Interestingly, in the present study, the Tetris (ACT) group showed improvements in PS and EF, contradicting the results of some prior studies [19]. It is important to note that NF may have contributed to the novel enhancements observed for the Tetris group in our study. These results suggest, as in previous studies [31], that performance feedback may be useful for optimizing cognitive training programs and thus resulting in superior improvements in cognitive functioning. Thus, we add to the body of work suggesting that CT-NF may provide additional cognitive benefits over and above CT or NF alone. 

It is also noteworthy to highlight that our findings are especially strong for a few reasons. One is that the chosen ACT was rigorous. The Tetris group, similar to the treatment (ABC group), underwent a live, virtual training, and participants in the ACT were asked to monitor and log their brain activity while playing, thus providing an additional NF component. Participants of the ACT group were provided with nearly equal treatment, and both groups reported similarly strong user-experience (UX) satisfaction ratings with the CT-NF program (ABC: M = 7.93, SD = 1.17; Tetris: M = 8.00, SD = 1.18). This confirms that any differences observed between the two groups (ACT vs. Tetris) were due to the actual CT component, and not liking or user-friendliness of each of the CT conditions. Moreover, our sample included many individuals that had never used an electronic tablet prior to the study. Previous studies with negative findings for Tetris included undergraduate student participants who may not have derived novelty effects, which the older participants in our study may have. Again, this highlights the rigor of the ACT in the present study. As such, it is not surprising that significant inter-group differences were not obtained despite the constellation of scores, inspection of figures, and the results of the composite cognitive scores; clearly indicating that the ABC group benefited from greater cognitive enhancements after treatment in comparison to the Tetris group.

### Future Directions and Limitations

Although the present set of findings are promising and novel, they are not without limitations. For example, the remote nature of our study, due to COVID-19 restrictions, served as both a strength and a weakness of the protocol. Although having our participants complete their CT at home increased the real-world applicability and replicability of our results, it also increased potential confounding factors due to the uncontrolled environmental setting. Moreover, although all participants received standardized training, due to the live and interactive nature of the study, there were some variability across training classes. Nevertheless, the user-satisfaction ratings, which were fairly high and similar for both groups, confirmed that there were not any significant between-group differences with respect to the users’ experience. Moreover, there may have been some variability in the placement of the brain sensor for each individual, despite this being included in the training curriculum, thus affecting the brain activity results. However, given that we took multiple measurements of brain activity (mean of about 23), any variations were likely to regress towards the mean, and only mean brain activity was used in our analyses.

An additional constraint of the study originated from the use of fNIRS. Although advantageous in many regards, fNIRS is limited by its own penetration depth, which allows for the detection of brain signals only from the cerebral cortex. Brain areas such as the hippocampus or entorhinal region, which are well-established for their role in memory and Alzheimer’s disease AD, cannot be detected by fNIRS. However, prior literature shows evidence of the association between activation in certain cortical regions, detectable by fNIRS (such as the dorsolateral and ventrolateral PFC [35]), and the success of memory encoding and retrieval. This gives us confidence that it is possible to detect hemodynamic changes related to memory and other aspects of cognitive function in PFC using fNIRS technology. 

In spite of all other controls, variability in efficacy of CT-NF programs may also be explained by individual differences in motivation, learning strategies, and responsiveness to the intervention. Moreover, given that our results culminated from follow-up immediately following the intervention protocol, future studies may want to assess whether cognitive enhancements are sustained, and if so for how long. Finally, future studies might also test the efficacy of a shorter training program, as differential trends in brain activity and games scores in our data suggest that performance may peak before the current protocol’s 4-week training period. In spite of these limitations, it is important to highlight the novelty of the study and the strength of the findings showing improved cognition in a healthy, aging individuals resulting from participation in a home-based, cognitive training programs with neurofeedback.

## 5. Conclusions

The results of the present study suggest that 4-weeks of CT-NF with ABC exercises result in enhanced cognition among older participants, specifically in measures of memory, verbal memory, processing speed, and executive function. Novel results showed that the ACT (Tetris) group also derived cognitive benefits from CT-NF such that participants displayed improvements in executive function and processing speed. In line with previous studies, we found that the strength of r. PFC brain activity during CT-NF (with ABC exercises) was associated with both cognitive (pre- and post) and game performance. In sum, this study suggests that CT-NF involving app-based exercises may be useful in promoting cognitive enhancements in older individuals. 

## Figures and Tables

**Table 1:** Sociodemographic characteristics of the sample by group (treatment vs. control).

Characteristic	ABC (*n* = 50)		Tetris (*n* = 36)	
	Mean	SD (Range)	Mean	SD (Range)
Age	68.56	6.80 (56–84)	63.36	5.61 (54–75)
	*n*	%	*n*	%
Gender (female/male)	36/14	71/29	29/7	81%/19%
Education (highest level)				
some college	7	14	7	19
Associated degree	3	6	2	6
Bachelor’s degree	23	46	7	19
Master’s degree	13	26	13	36
Professional degree	1	2	4	11
Doctorate	2	4	2	6

Overview of demographics of the study sample. Table 1 depicts the mean age, gender, and education of study participants for the treatment (ABC) and control (Tetris) groups.

**Table 2:** Pre- and post-cognitive assessment results for the ABC (treatment) group.

	Pre ABC Scores		Post ABC Scores				
	*M*	*SD*	*M*	*SD*	*t* (48)	*p (Two-Tailed)*	*p (One-Tailed)*
Composite Score	304.59	36.52	317.48	34.78	2.60	0.01 *	0.006 **
Memory	92.94	7.69	95.10	8.21	1.77	0.08	0.04 *
Verbal memory	49.89	5.17	51.35	5.75	1.99	0.05 *	0.03 *
Visual memory	43.05	4.60	43.75	4.46	1.04	0.30	0.15
Processing speed	46.35	8.98	47.99	8.98	1.86	0.07	0.03 *
Executive function	35.11	12.99	42.54	10.04	5.25	0.000 **	<0.001 **
Working memory	9.07	4.73	9.42	4.72	0.66	0.52	0.26
Sustained attention	28.18	7.17	29.20	6.43	1.41	0.17	0.09

Results of pre- and post-cognitive assessments for the treatment (ABC) group are shown. Paired samples *t*-tests were conducted to compare pre- and post-cognitive assessments. *t*-values and *p*-values (of statistical significance) for one-tailed and two-tailed tests are provided above. Significant results were labeled with asterisks: * (*p* < 0.05), ** (*p* < 0.01).

**Table 3:** Pre- and post-cognitive assessment results for the Tetris (ACT) group.

	Pre Tetris Scores		Post Tetris Scores				
	*M*	*SD*	*M*	*SD*	*t* (32)	*p* *(Two-Tailed)*	*p (One-Tailed)*
Composite Score	315.04	48.73	318.95	53.70	0.68	0.50	0.25
Memory	95.41	12.76	93.98	14.40	0.23	0.82	0.41
Verbal memory	51.82	6.41	51.00	7.63	0.25	0.80	0.40
Visual memory	44.14	5.71	42.97	7.80	0.59	0.56	0.28
Processing speed	45.69	9.43	48.21	10.30	1.98	0.06	0.03 *
Executive function	37.57	18.25	42.39	13.57	3.15	0.004 **	0.002 **
Working memory	10.49	3.71	10.28	3.74	0.16	0.87	0.44
Sustained attention	29.92	6.39	30.13	6.77	.13	0.90	0.45

Results of pre- and post-cognitive assessments for the control (Tetris) group are shown. Paired samples *t*-tests were conducted to compare pre- and post-cognitive assessments. *t*-values and *p*-values (of statistical significance) for one-tailed and two-tailed tests are provided above. Significant results were labeled with asterisks: * (*p* < 0.05), ** (*p* < 0.01).

**Table 4:** Change scores and results from ANCOVAs examining between group differences in cognitive function change scores.

	ABC		Tetris			
	*M*	*SE*	*M*	*SE*	*F*	*p*
Composite Score	12.95	5.42	6.02	8.88	0.15	0.70
Memory	2.87	1.61	−0.72	3.07	0.17	0.69
Verbal memory	1.96	0.98	−0.39	1.57	0.06	0.82
Visual memory	1.18	0.87	−0.92	1.53	0.46	0.50
Processing speed	1.75	1.01	2.82	1.42	0.00	0.99
Executive function	6.78	1.25	5.31	1.69	0.11	0.74
Working memory	0.34	0.56	−0.11	0.64	0.02	0.88
Sustained attention	1.20	0.86	−0.15	1.17	0.01	0.94

## Data Availability

No analyses in the present research are redundant with any published findings.
